# *Toxoplasma gondii* in the Eurasian kestrel (*Falco tinnunculus*) in northern Italy

**DOI:** 10.1186/s13071-020-04134-x

**Published:** 2020-05-19

**Authors:** Tiziano Iemmi, Alice Vismarra, Carlo Mangia, Rolando Zanin, Marco Genchi, Paolo Lanfranchi, Laura Helen Kramer, Nicoletta Formenti, Nicola Ferrari

**Affiliations:** 1 Department of Veterinary Science, University of Parma, Parma, Italy; 2 URCA (Unione Regionale Cacciatori dell’Appennino), Parma, Italy; 3 Department of Veterinary Medicine, Università degli Studi di Milano, Milan, Italy; 4 Istituto Zooprofilattico Sperimentale della Lombardia e dell’Emilia Romagna “Bruno Ubertini”, via Bianchi 7/9, 25124 Brescia, Italy; 5 Centro di Ricerca Coordinata Epidemiologia e Sorveglianza Molecolare delle Infezioni, EpiSoMI, Università degli Studi di Milano, Milan, Italy

**Keywords:** Biodiversity, Diet influence, Kestrels, *Toxoplasma gondii*, Wildlife

## Abstract

**Background:**

Identifying factors that sustain parasite transmission is important for understanding their spread and emergence, including how changes in biodiversity may affect parasite prevalence and spread. *Toxoplasma gondii* is a protozoan parasite infecting humans and animals. Birds can acquire *T. gondii* infection through ingestion either of oocysts from the ground or of tissue cysts present in infected prey and are therefore suitable indicators of the presence of *T. gondii* in the natural environment.

**Methods:**

The aim of the study included the evaluation of *T. gondii* seroprevalence in clinically healthy Eurasian kestrels (*Falco tinnunculus*) using a modified agglutination test. Birds were captured in a small area of Parma (northern Italy) for two consecutive years (2016–2017), sex and age determined and serological study carried out. Food sources for the birds were also evaluated, in particular rodent and grasshopper population estimates in the study area. The biomass of rodents and grasshoppers per hectare was estimated in order to directly compare food availability. Statistical analyses were performed in order to evaluate factors influencing the probability of kestrels being *T. gondii-*seropositive using R 3.4.4 fitting linear mixed-effect models with the ‘glmer’ function of the package *lme4*, ‘lsmean’ in package *lsmean* for pair-wise post*-*hoc comparisons using differences of least square means (DLSM) and the ‘betareg’ function of the package *betareg* for beta regression.

**Results:**

Seroprevalence for *T. gondii* was 33.3% (49/147) in 2016, while in 2017 seroprevalence decreased to 14.3% (13/91). An increase in the probability of kestrels being *T. gondii-*seropositive was associated with a higher rodent biomass in the environment, suggesting a positive feedback of the biotic factors driving infection risk.

**Conclusions:**

These results underline the need for multidisciplinary studies aimed at better understanding pathogen-host relationships and for predictions in disease ecology.
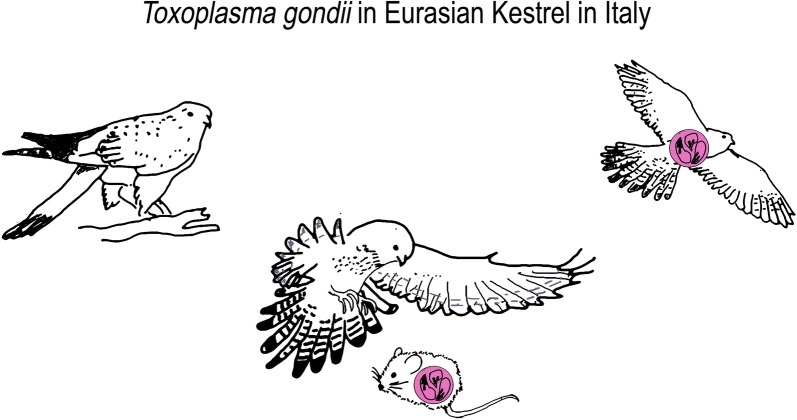

## Background

Understanding factors that sustain parasite transmission is a key step in our comprehension of their spread and emergence. Recent global changes in the environment have led to a growing attention on the impact of biodiversity on parasite transmission, suggesting that loss in biodiversity can determine a rise in pathogen circulation [1]. Indeed, the presence of multiple animal species can support the dilution of infective stages in diverse organisms with different infection susceptibility. For food-borne parasites, this implies that a diversified diet provides a decreased exposure to infection compared to one focused on those species with higher prevalence.

*Toxoplasma gondii* is protozoan parasite with a complex life-cycle. Tissue stages can be found in all warm-blooded species [2] and the parasite circulates in a wide range of environments, including marine and mountain ecosystems [3, 4]. Although the definitive hosts are represented by felids that release and spread oocysts into the environment, *T. gondii* can be efficiently transmitted even between intermediate hosts, allowing its persistence in the absence of definitive hosts [5, 6]. Indeed, through consumption of infected tissues, this parasite can be transmitted along the food chain, leading to bio-amplification.

Birds can acquire *T. gondii* infection through ingestion of either sporulated oocysts found in the environment or through ingestion of cysts present in the tissue of infected prey. In particular, carnivorous birds are important indicators of *T. gondii* prevalence in prey species [7] and birds of prey and scavenger species are likely more exposed to *T. gondii* infection compared to herbivorous birds, due to the phenomenon of accumulation of the parasite along the food chain [2]. Diet of predatory birds and abundance of prey species will influence the prevalence of *T. gondii* infection. Interaction at the wild-domestic interface, typical of many habitats including those where birds of prey are present, likely plays a role in the epidemiology of *T. gondii*.

*Toxoplasma gondii* infection may also cause disease in wild bird species and this will depend on the host’s susceptibility, physical condition [8], and the infective stage/genotype of the parasite [8, 9]. Clinical signs associated with infection include neurological, ocular and pulmonary disease or multi-organ involvement [9]. The aim of the present study was to determine how the environmental changes of the structure of prey populations can be reflected on *T. gondii* seroprevalence in a predator species. In the present study, we focused our attention on the Eurasian kestrel (*Falco tinnunculus*), a predator of small vertebrates (birds, mammals and reptiles) and arthropods [10], thus representing a potentially reliable bioindicator for the presence of *T. gondii* [7]. Furthermore, kestrels are a protected raptor species that has enjoyed good recovery in terms of population in recent years in northern Italy. This has led, however, to an increasing number of bird strikes at different airports in the area and the need for bird strike mitigation activities. This allowed the sampling in 2016 and 2017 of a large number of birds during capture-release projects.

## Methods

### Animals and trapping

The study was carried out within the context of a bird-strike hazard control project at the “Giuseppe Verdi” International Airport in Parma (located in the middle of the Po Valley, northern Italy, 44°49′20″N, 10°17′43″E). The study area included the airport surface, 92 ha of meadow grass located next to cultivated fields and close to the urban territory of Parma. The project consisted of the daily capture of kestrels present at the airport site, identification with numerated tarsal rings and the subsequent relocation and release of subjects to an area approximately 18 km (or 40 km in case of re-capture) away. Birds were captured employing 10 “Swedish goshawk” traps, modified for use with kestrels [11].

The captures were conducted from June 2016 to December 2016 and from May 2017 to December 2017. The periods were defined by the airport management company (SOGEAP s.p.a.) based on the period of greatest kestrel bird strike risk, i.e. late spring (by young fledglings) to early winter (greater dispersion of birds).

Identification of species, sex and age was performed according to Village [10] and Christie & Ferguson-Lees [12]. Briefly, birds were considered young when complete juvenile plumage was present and no mutated flight feathers were seen and in general they are the subjects born in the spring of the current year and therefore with an age less than one year of life. Sub-adults were the subjects born in the spring of the previous year, characterized by a changing juvenile plumage, with adult feathers in eruption. Birds with mature plumage and complete chromatic patterns (complete after the second year of life, to reaching sexual maturity) were considered adults. Sex determination of adults and sub-adults was based on typical plumage dimorphism. Young birds were sexed based on morphometric measurements (body weight, tarsal length and beak tip to nape distance) [10, 12].

During the study period, food sources for the birds were also evaluated, in particular rodent and grasshopper population estimates in the study area. The capture/marking/recapture method was used for rodents, employing 30 traps (10 Sherman and 20 Ugglan traps), considering as proxies of rodent abundance the Peterson index [13, 14]. Grasshopper enumeration was performed according to Ausden & Drake [15], with direct observation and count, by walking along a transect and counting the insects that jumped a step of a meter in front of the operator. Evaluation of abundance for both rodents and grasshoppers was performed twice a year, in mid-July/mid-August and in October, by the same author (TI) for the entire study period in order to guarantee comparability of results. Species, age class and sex determination of rodents was performed in according to Bjärvall & Ullström [16].

The biomass of rodents and grasshoppers per hectare was estimated in order to directly compare food availability. In particular biomass was calculated by scaling the number of rodents and insects per hectare by the average weight of the sampled individuals of these species. Diet composition was evaluated by examination of regurgitated pellets composition (consisting of the indigestible remains of prey) collected from transport boxes following the technique described by Yalden [17].

### Sampling and release

All captured kestrels were brought to the laboratory and evaluated for biometrical measurements, marked with a numerated tarsal open ring and when possible, approximately 1 ml of blood was drawn from the right jugular vein and stored in heparinized 1 ml tubes. Subsequently, serum was obtained by centrifugation for 5 min at 1500 *rpm*. All serum samples were stored at − 20 °C until serological analysis.

Birds were subsequently relocated to appropriate release areas; birds from the first capture were relocated approximately 18 km away, while birds from the second and third captures were released at a distance of 40 km from the trapping area. The tarsal ring application allowed individual recognition in the event of re-capture and avoided testing the same subject multiple times.

### *Toxoplasma gondii* serology

A commercial modified agglutination test (MAT) kit (Toxo-Screen DA^®^; BioMerieux SA^®^, Marcy-l’Etoile, France) was used for *T. gondii* serology following the manufacturer’s instructions. All samples were tested at a dilution of 1:25, as already reported by other authors [18, 19] for serum samples analyses in birds, and this value was also the cut-off we considered to discriminate between negative and positive animals [18–20]. In each analytical session, positive and negative controls of the commercial kit were used.

### Statistical analysis

Factors influencing the probability of kestrels being *T. gondii*-seropositive were analyzed through a generalized linear model with binomial distribution, considering the serological status (positive/negative) as a response variable and as explanatory variables, year of capture, sex and age classes.

Variation in prey abundance between years was tested using Studentʼs t-test.

Finally, kestrel diet preference was inspected through beta regression, considering as a response variable the proportion of pellets containing a prey category (small mammals *vs* insects) and the identity of the prey category, its environmental biomass abundance and that of the alternative prey category, as explanatory variables. The relationship between serological status and food availability, was evaluated through a linear mixed effect model with binomial distribution, considering the serological status as a response variable and the biomass of rodents and insects during the capture period as explanatory variables. Year was included as random intercepts in order to take into account temporal variability.

For all models we initially fitted a maximal model including all biological meaningful first order interactions, and through a likelihood-ratio test, the minimal adequate models were obtained, by dropping those factors that do not contribute significantly to explain the variability of the response variable. For the linear mixed effect model this analysis was first carried out on the random term structure and subsequently on the fixed terms [21].

The analyses were performed using R 3.6.3 [22] fitting linear mixed-effect models with the ‘glmer’ function of the package *lme4*, ‘lsmean’ in package *lsmean* for pairwise *post-hoc* comparisons using differences of least square means (DLSM) and the ‘betareg’ function of the package *betareg* for beta regression. The significant threshold was *P* < 0.05.

## Results

### Animals and capture

In 2016, 343 kestrels were captured, measured and released; 312 were young, 12 were sub-adults and 19 were adults (155 males/188 were females). Of these, 147 (63 males and 84 females) were tested for *T. gondii* by serology, including 116 young, all sub-adults (*n* = 12) and all adults (*n* = 19). In 2017, 91 kestrels (42 males and 49 females) were captured, of which 70 young, 6 sub-adults and 15 adults. All were tested for *T. gondii* antibodies.

### *Toxoplasma gondii* infection prevalence

**Fig. 1:**
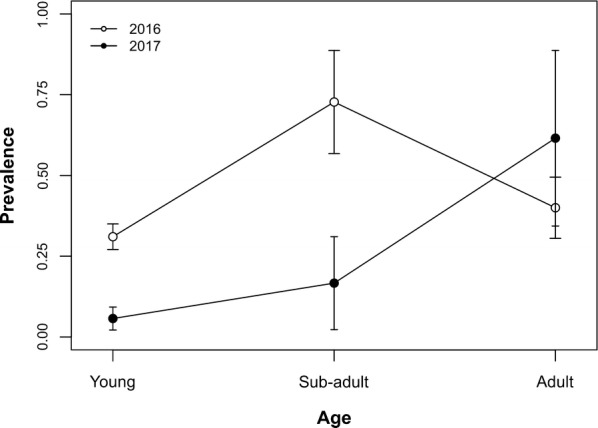
*Toxoplasma gondii* seroprevalence (± 95% CI) of Eurasian kestrel in 2016 and 2017 broken down by age class

Table 1 reports *T. gondii* seroprevalence in kestrels captured during the two study periods. Kestrels had an overall prevalence of 26.1% (95% CI: 20.5–31.6%) which showed a temporal variation between the two years. A significantly higher percentage of birds was seropositive in 2016 compared to 2017 (Tables 1, 2). Age influenced the probability of being seropositive (Fig. 1). Age class differences were observed in 2016, but they were not statistically significant (*P* = 0.53), while in 2017 young kestrels had significantly lower prevalence values compared to adults captured during the same year (*P* < 0.001). Interestingly, adult birds captured in 2017 had comparable values to sub-adults in 2016 (*P* = 0.99) (Table 1). No differences in seroprevalence based on sex (male *vs* female) were observed for either year of capture.

**Table 1:** Serology results for *T. gondii* antibodies in kestrels from Italy, captured in 2016 and 2017

	Age class			
	Young	Sub-adult	Adult	Total
2016				
Sampled animals	116	12	19	147
Prevalence % (95% CI)	29.3 (21.2–37.6)	58.3 (30.4–86.2)	42.1 (19.9–64.3)	33.3 (25.7–40.9)
2017				
Sampled animals	70	6	15	91
Prevalence % (95% CI)	5.7 (0.3–11.1)	16.6 (0–46.5)	53.3 (28.1–78.6)	14.3 (7.1–21.5)

**Table 2:** Minimum generalised linear model of the effects of host characteristics and environmental variables on *T. gondii* prevalence in kestrels

Variable	Coefficient	Deviance	*df*	*P*-value
Year		12.77	1	< 0.001
2016	0.00			
2017	− 2.00			
Age class		15.95	2	< 0.001
Young	0.00			
Sub-adult	1.777			
Adult	0.39			
Year: Age class		12.17	2	< 0.001
2017: Sub-adult	− 0.58			
2017: Adult	2.88			

*Abbreviation*: *df*, degrees of freedom

### Prey abundance, kestrel diet and effects on *T. gondii* infection

**Fig. 2:**
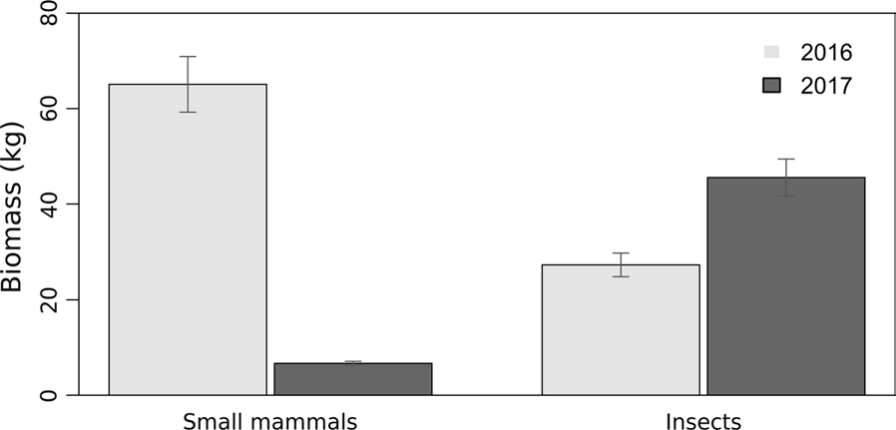
Environmental biomass of small mammals and insects (± 95% CI) in 2016 and 2017

The rodent species captured in the area included *Apodemus sylvaticus* and *Microtus savii*. Prey biomass differed between the two years considered. Rodent abundance in 2017 was significantly lower compared to 2016 (*t*_(147)_ = 19.5, *P* < 0.001), while grasshoppers showed an opposite trend (*t*_(158)_ = 7.7, *P* < 0.001; Fig. 2).

**Fig. 3:**
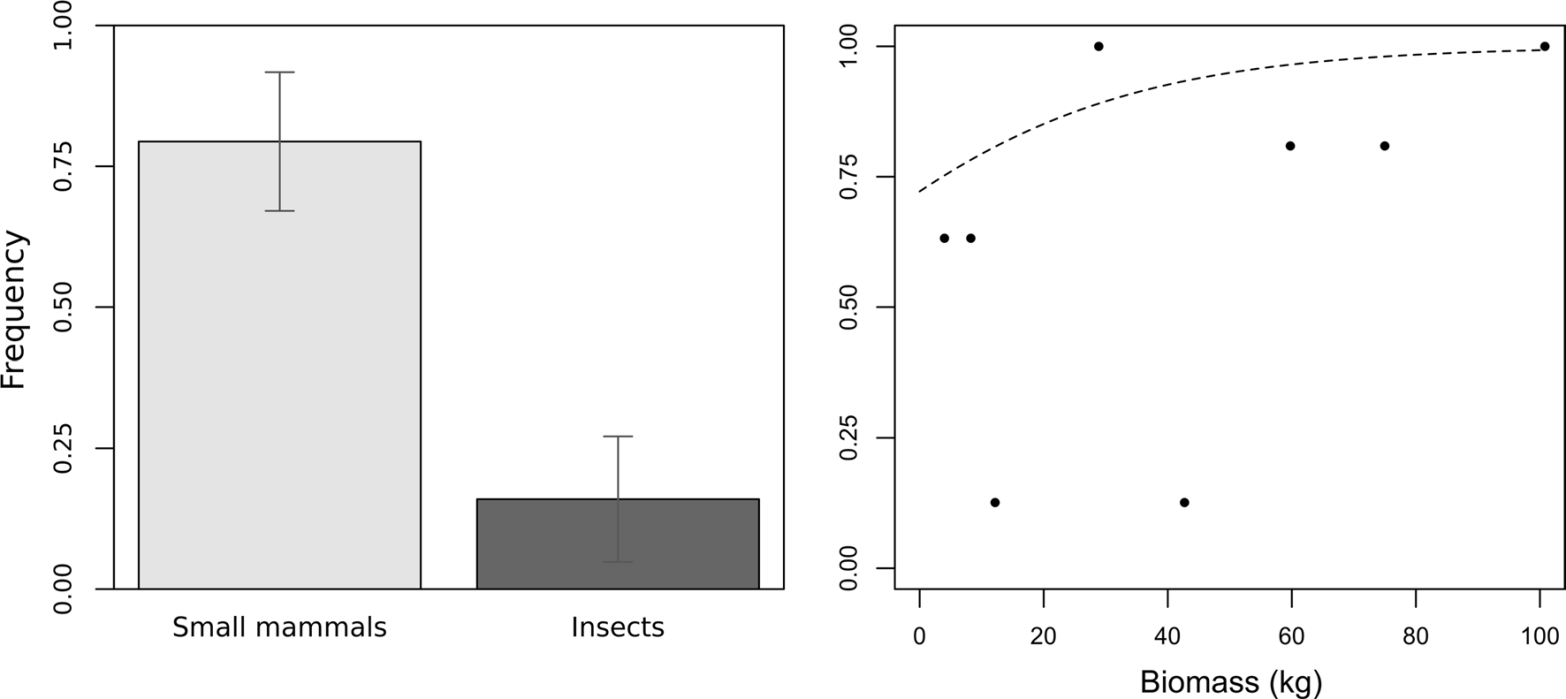
Kestrel pellets composition according to prey category (**a**) and the environmental biomass of the prey category (**b**)

Overall, kestrel diet was contained significantly more rodents compared to insects (beta regression coeff. = 3.0, *Z* = 3.91, *P* < 0.001) (Table 3, Fig. 3a). In 2016, 154 kestrel pellets were examined and 100% contained rodent remains; of these, 87.4% contained only rodent remains while the remaining 12.6% were composed of a mixture of rodent remains and insect exoskeletons (primarily orthopterans). Out of the 68 kestrel pellets examined in 2017, only 19.1% contained solely rodent remains, while 44.1% were made up of rodent and insect exoskeleton remains, and 35.3% contained only insect exoskeletons. Moreover, the proportion of prey species in the pellet was associated with their environmental biomass values (beta regression coeff. = 0.04, *Z* = 3.45, *P* < 0.001) (Fig. 3b).

**Table 3:** Beta regression minimal model of the effects of factors influencing pellets kestrel composition

Variable	Coefficient ± SE	*Z*-value	*P*-value
Rodent	3.00 ± 0.76	3.91	< 0.001
Species biomass	0.04 ± 0.01	3.45	< 0.001

**Fig. 4:**
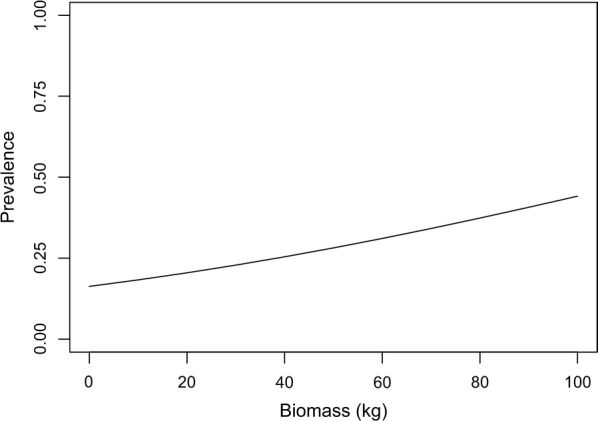
Effects of biomass of small mammals on kestrel prevalence estimated from the generalized mixed model

Finally, the probability of kestrels being *T. gondii-*seropositive increased with the biomass of rodents in the environment (*χ*^2^ = 15.09, *P* < 0.001) (Fig. 4), while insect biomass did not show any effect (data not shown).

## Discussion

Results of the present study suggest that European birds of prey are suitable indicators of *T. gondii* circulation in the habitat where these birds live and reproduce, in agreement with previous reports [20, 23, 24].

The Eurasian kestrel is one of the most common birds of prey in Europe, occupying a wide range of habitats, and is among the most common aerial predators in many European cities [25]. In the present study 47.7% of adult birds examined were positive to *T. gondii* circulating antibodies. Previous studies in Europe have reported values ranging from 5.5% [26] to 30% [23]. Several other animal species have been indicated as indicators of environmental contamination with *T. gondii*, including wild boar and free-ranging pigs, which are both prevalent in the region where the present study was carried out. Thus, Gazzonis et al. [27] reported 43% of wild boars were positive on *T. gondii* serology, while Bacci et al. [28] reported a 95% prevalence in free-ranging pigs.

Age has been defined as an important risk factor for *T. gondii* infection in many animals, including birds [2]. In the present study, statistically significant age-associated changes in seroprevalence were also observed in kestrels (considering data collected for two seasons). Young kestrels had lower apparent seroprevalence than adult birds. This is likely due to the cumulative exposure of birds as they grow older [7].

The seroprevalence of *T. gondii* was lower in 2017 compared to 2016 and sub-adults from 2016 had comparable values with adults from 2017, suggesting that this age cohort maintained the infection prevalence and exposure to infection did not increase between the two years. Moreover, the drastically lower values in young birds in 2017 would indicate that sources of primary infection for these birds were scarce. Indeed, there was wide variation in the kestrels’ diet between the first and second year, associated with a lower seroprevalence for *T. gondii* in 2017. The decrease in rodent population observed in 2017 led to a shift toward insects as a food source; results suggest that kestrels do not select their prey category but feed on the most abundant preys. While rodents are an essential source of *T. gondii*, insects are only potential mechanical vectors for oocysts [29]. The present study indicates the role of rodents as a primary source of *T. gondii* infection in birds of prey, with significant association of their environmental abundance with *T. gondii* infection prevalence in kestrels. It is also worth noting that the MAT is regarded as the most sensitive and specific test for toxoplasmosis in birds. Although there is no validation of MAT for kestrels, viable *T. gondii* has been isolated from feral chickens with a MAT titer of 1:5 [30]. Thus, the 1.25 dilution as a positive cut-off used in the present study may have underestimated the true prevalence.

Therefore, we suggest that the biodiversity of the principal prey species in the geographical area studied had a direct effect on *T. gondii* prevalence in kestrels. Similar results were reported by Johnson et al. [31] in a study aimed at identifying risk factors for *T. gondii* infection in sea otters, where the authors reported that otters that chose marine snails were more likely to be infected with *T. gondii* compared to otters from an area rich in abalone. This would suggest, as in the present study, that dietary habits that cope with limited food resources may lead to a change in infection rates with *T. gondii*. Likewise, a recent seroprevalence study on African lions, spotted hyenas, striped hyenas and bat-eared foxes (the latter of which eats insects) highlighted that the risk for toxoplasmosis is higher in carnivores compared to insectivorous carnivores (bat-eared foxes) [32]. The same was reported for carnivorous birds and carnivorous mammals when compared to herbivorous mammals (as Camelidae and Equidae) [33].

## Conclusions

To the best of our knowledge, the present study is the first to evaluate the association between prey abundance and *T. gondii* infection in the European kestrel. Results from the present study would suggest that loss of natural habitat and the decrease of prey species, may lead to feedback on the biotic factors driving infection risk. These results underline the need for multidisciplinary studies aimed at better understanding host-pathogen relationships and for predictions in disease ecology.

## Data Availability

All data generated or analysed during this study are included in this published article.

## Abbreviations

MAT: Modified agglutination test
DLSM: Differences of least square means
ENAC: Italian Authority for the management of civil aviation (Ente Nazionale Aviazione Civile)
